# Characterizing the quality-of-life impact of Duchenne muscular dystrophy on caregivers: a case-control investigation

**DOI:** 10.1186/s41687-021-00386-y

**Published:** 2021-11-20

**Authors:** Carolyn E. Schwartz, Roland B. Stark, Ivana F. Audhya, Katherine L. Gooch

**Affiliations:** 1 DeltaQuest Foundation, Inc, 31 Mitchell Road, Concord, MA 01742 USA; 2 Departments of Medicine and Orthopaedic Surgery, Tufts University Medical School, Boston, MA USA; 3 Sarepta Therapeutics, Cambridge, MA USA

**Keywords:** Duchenne muscular dystrophy, Impact, Caregivers, Quality of life, Resilience

## Abstract

**Background:**

This study examined the impact of Duchenne muscular dystrophy (DMD) on family-member caregivers in terms of quality of life, life stress, and indirect costs, as compared to a stratified comparison group of parents of similar-age children without DMD.

**Methods:**

A web-based survey included DMD caregivers and a nationally representative comparison group of parents of children without DMD stratified by Child Age Group. Outcomes included quality of life, resilience, caregiver impact, stressful life events, financial strain, out-of-pocket expenditures, work productivity and unrealized ambitions. General linear models assessed the main effect of Caregiver Group and the interaction of Caregiver Group with Child-Age-Group, after adjusting for demographic covariates.

**Results:**

Compared to parents without a DMD child, DMD Caregivers reported better physical health but worse mental health, positive affect/well-being, environmental mastery, difficulty paying bills, and more hours missed from work. Providing caregiving support for DMD teenagers was the most challenging. DMD caregivers curtailed their educational and professional ambitions, and modified their homes to accommodate the disability associated with DMD. Their non-DMD children had to make sacrifices as well. Nonetheless, in resilience and life stress, DMD caregivers were comparable to the comparison group, and showed consistent levels of positive emotions across the age of their DMD child.

**Conclusion:**

DMD caregivers fared worse on most outcomes and faced more hurdles in work life. They face constraints and hidden costs that impact their health and financial well-being. Caregivers of teenagers with DMD reported higher impact. Nonetheless, parents of DMD children of all ages maintained notable resilience and positivity.

**Supplementary Information:**

The online version contains supplementary material available at 10.1186/s41687-021-00386-y.

## Introduction

Taking care of others is a common part of adult life, particularly as parents. For many, helping others benefits their own health-related quality of life (HRQOL) [1], providing a greater sense of purpose in life [2, 3], enhancing mental health [4, 5] and, among females, enhancing physical health [5, 6]. When helping others becomes less occasional and develops into a time- and energy-demanding long-term role, then the “caregiving” can become a burden. Providing caregiving support to aging parents, sick spouses/partners, or chronically-ill children has been reported to have direct and deleterious effects on the caregiver’s health and work productivity [7–12]. Caregiver burden in a range of progressive neurologic diseases has been found to be associated cross-sectionally with the patient’s cognitive and executive-functioning status [13, 14] and sleep problems [15], and longitudinally with the patient’s anxiety and depression [16–18].

While there are general truths about the challenges of chronic caregiving, each caregiving context has its unique challenges. For Duchenne muscular dystrophy (DMD), the challenges of caregiving rise above the challenges of usual parenting and likely relate to the early age of diagnosis, progressive disease trajectory, life domains affected, and truncated life expectancy. DMD is a progressive, rare, and irreversible neuromuscular disorder occurring primarily in males -- 1 in 5050 live births [19–21]. Usually diagnosed by age 5, the disorder presents as delayed development that includes motor difficulties [22], and may include cognitive impairment and attention-deficit disorders [23]. On average by age 10–12, progressive muscle weakness leads to individuals’ loss of ambulation, upper-limb function problems, and comorbid conditions such as scoliosis and muscular contractures [22]. By age 15, individuals often experience increased difficulty breathing and life-threatening heart and lung conditions [24]. People with DMD face profound uncertainty regarding lifespan, typically dying in their 20s to early 30s [24], although medical advances [19] have led to longer life expectancies [25].

A number of published articles have addressed the impact of DMD on family-member caregivers [26] including healthy siblings [27, 28]. Caregiving demands increase with DMD progression [29] and low patient vitality and mental health [30], and this demand impairs multiple domains of caregivers’ health-related QOL [26, 30], including sleep quality [31], mental health [11, 32, 33], social [34] and sexual functioning [35], and work productivity [36]. Although these studies provide helpful descriptive information, they are primarily cross-sectional ‘snapshots’ of impact, many using generic tools that may have missed important domains and aspects of impact specific to DMD. Further, only one of 25 papers examined included a comparison group, and the sample sizes were generally small [31]. The literature has not reported caregiver impact by the different phases (i.e., stages) of DMD separately in estimating impact. To study the impact of DMD caregiving fully, one needs to ensure robust sample sizes across age groups that reflect disability progression over time. In this way, caregiver impact can be investigated separately by each phase or stage of DMD.

The present study is descriptive and seeks to address the abovementioned gaps in the literature. It examines the impact of DMD on family-member caregivers in terms of QOL, life stress, and indirect costs. DMD caregivers are compared to a stratified group of parents of similar-age healthy children.

## Methods

### Sample and procedure

This study recruited DMD-caregiver participants via Rare Patient Voice (www.rarepatientvoice.com), patient-advocacy groups, and the snowball technique [37] (i.e., word of mouth). Comparison-group participants were recruited via IPSOS-Insight, LLC (www.ipsos.com), and were selected to accurately represent the United States in age, race/ethnicity, gender, and region. Eligible participants were age 18 or older, able to complete an online questionnaire, and (for DMD caregivers only) providing caregiving support to a family member with DMD at least two years old.

Telephone interviews were conducted with 15 DMD parent-caregivers to pretest and adapt possible measures for content validity and to develop items assessing out-of-pocket expenditures and impact on siblings (described in Measures below). These interviews shared initial proposed questionnaires as web-based surveys for the interviewee to complete prior to the interview. The interviewer then asked in the interview whether these questionnaires addressed relevant topics. The questionnaires were then modified and pre-tested with other interviewees, with similar content-validity queries. The final set of questionnaires addressed suggestions and concerns raised by the interviewees. The subsequent web-based survey was administered June through November 2020 through the HIPAA-compliant, secure Alchemer engine (www.alchemer.com).

Recruitment was stratified by Child Age Group: 2–7, 8–12, 13–17, and >= 18, reflecting the above-described disability trajectory [22–25]. Although DMD can progress at varying rates, these age strata reflected the phases of DMD progression: ambulatory phase (up to age 7), transitional phase (up to age 12), and non-ambulatory phase (age >= 13), with increasing dependence and involvement of other systems into adulthood (age >=18). If caregivers were providing caregiving support for more than one person with DMD, they were asked to report on the eldest or most disabled (the “index patient”). Participants with motor, visual, and/or other problems that made it difficult for them to complete the web-based survey instrument enlisted the assistance of a household member to enter their answers. Caregivers were paid $75 honoraria to compensate them for their time completing the survey. The protocol was reviewed and approved by the New England Independent Review Board (NEIRB #20201623), and all participants provided informed consent prior to beginning the survey.

### Measures

Unless otherwise noted, the measures described below were collected from all study participants. A subset of measures that were relevant only to DMD were collected only from DMD caregivers.

*Quality of Life* was assessed using a battery of brief, standardized tools. The PROMIS-10 General Health is a ten-item measure that yields scores for physical and mental health [38]. The NeuroQOL Positive Affect and Well-Being is a 9-item measure of well-being [39]. The Ryff Environmental Mastery is a 7-item subscale of the Ryff Psychological Well-Being measure that assesses how well the individual feels able to deal with the demands of her/his environment [40]. The Centers for Disease Control (CDC) Healthy Days Core Module [41] was used to operationalize resilience (see Statistical Analysis below). In this measure, two items ask the respondent to indicate how many days of the past 30 days their physical health (Physical Health Problems) or mental health (Mental Health Problems), respectively, was not good. A third item, Activities of Daily Living Impaired (ADL Impaired) asks how many days of the past 30 the respondent’s poor physical or mental health kept them from doing their usual activities, such as self-care, work, or recreation.

*Caregiver impact* was assessed only in the DMD caregivers, using a DMD adaptation of the Hemophilia Caregiver Impact measure [42, 43]. Items were modified to reference DMD and were pretested for content via the telephone interviews. The resulting 39-item DMD Caregiver Impact Measure (DCI) includes seven negative-impact subscales (Practical, Physical, Financial, Symptom, Lifestyle, Social, and Emotional) and one positive-impact subscale (Positive Emotions).

*Life stress* was measured using a subset of items from the Urban Life Stress Inventory [44, 45]. The present study included a list of 18 areas of life, and the respondent was asked to indicate how much stress s/he experienced during the past 12 months using a five-level rating scale (no stress to extreme stress).

*Financial strain* was measured by an item asking about the respondent’s difficulty paying bills [46]. Research to date suggests that this item yields fewer missing values than a question about household income [46] and is a better indicator of financial well-being because it directly assesses ability to make ends meet [47].

*Indirect costs of DMD* were operationalized as out-of-pocket expenditures, work productivity, and unrealized ambitions. *Out-of-pocket expenditures*, assessed only in DMD caregivers, included eight home and vehicle modifications that might have been implemented to accommodate their child’s DMD. *Work productivity* was operationalized using an item about hours missed from work in the past week, from the Work Productivity and Activity Impairment measure [9, 48–50]. *Unrealized ambitions* were operationalized using statistical modeling (see below) that included the DeltaQuest Reserve-Building (DQRB) measure’s [51] Occupational Complexity Index and the respondent’s educational attainment. Occupational complexity was assessed using questions querying the job that was closest to the respondent’s current or past occupation, which were then scored for complexity using the Occupational Information Network (O*NET) system [52]. Under this comprehensive, in-depth job-classification system, scores range from low complexity [1] to high complexity [5]), with higher scores reflecting more training and skills required to perform that occupation [53]. Some caregivers were unemployed and/or never employed because of their caregiving responsibilities, and they were assigned an O*NET value of “2” reflecting their role as caregiver. An O*NET value of “2” is the score used for caregivers in the O*NET data base (e.g., certified nursing assistant, home health aide, nurse’s aid). Educational attainment summarized level of formal education, ranging from less than 12th grade to doctoral degree. Finally, *Impact on Siblings*, assessed only in DMD caregivers, queried nine rating-scale statements ranging from strongly disagree [1] to strongly agree [7] that related to sacrifices mentioned by caregivers in the above-mentioned interviews.

*Demographic characteristics* included year of birth, gender, race, ethnicity, education, marital status, weight, height, with whom the person lives (which yielded a count of people living in the household who could provide caregiving support), number of children, number of children with DMD (if applicable), smoking or vaping status, employment status, caregiver’s and index child’s comorbidities, source of referral to the study, and Zone Improvement Plan (ZIP) code. The comorbidities listed for the index child were selected on the basis of documented higher prevalence in people with DMD [22, 23].

*COVID-19 specific information* included whether anyone in the household was or had been infected with the novel coronavirus-2019, and rating scale items on COVID impact related to financial hardship, isolation, social support, and continuity of healthcare.

### Statistical analysis

Descriptive statistics were used to summarize concepts measured only in DMD caregivers. When concepts were measured in both groups, t-tests or chi-squared statistics were used to compare groups, and such concepts were also included in the general linear modeling.

Demographic differences between the two caregiver groups were controlled using propensity scores when we tested for differences in outcomes. A logistic regression model was computed predicting the dependent variable Group (DMD vs. comparison caregiver) from the following nine caregiver covariates: age, gender, race, Hispanic ethnicity, marital status, body mass index, number of people living in the household who could provide caregiving support, smoking/vaping status, and total number of children in the household. For 12% of participants, propensity scores based on the above were missing data and so were assigned in a subsequent model using the six predictors above that were missing no data.

To operationalize resilience, we built on a precedent for using residual modeling to infer resilience based on the behavior of other variables in the model [54–56]. This approach has been used in multiple studies of chronically-ill people and their caregivers [47, 57, 58]. The approach involves regressing the CDC Healthy Days ADL Impaired on Physical Health Problems, Mental Health Problems, and their interaction. The residuals from the regression model were saved and multiplied by negative one (− 1). Thus, a high resilience score reflects “over-performance”, or more days than expected that the respondent was able to function despite physical or mental health problems or their synergistic effect [57]. Accordingly, a low resilience score reflects “under-performance”, or fewer days than expected that the respondent was able to function despite such problems.

Group differences on outcomes were investigated using general linear modeling. To test the hypothesis that DMD caregivers had different outcomes than comparison caregivers, analysis-of-covariance models included as independent variables Group and propensity score and predicted the following dependent variables: QOL (physical and mental health, positive affect & well-being, environmental mastery), resilience, stress, financial strain, and time missed from work. A separate set of models built on the above by adding the variable Child Age Group (referent category: child age 2–7) and an interaction term of Group * Child Age Group. These latter models examined whether DMD caregivers’ predictive relationships were different from comparison caregivers’ as a function of the DMD index child’s age.

To operationalize Unrealized Ambitions*,* general linear modeling predicted the O*NET score (Occupational Complexity as dependent variable) from independent variables Group, Educational Attainment, their interaction, and the propensity score. In a subsequent model, Child Age Group and its interaction with Group were also included. Finally, an ordinal regression model was tested predicting caregiver educational attainment from Group and from ZIP-code-based population percentages of people who had vocational training/some college, a bachelor’s degree, and a graduate degree. This latter model compared the caregivers’ educational attainment to their communities’ norms.

IBM SPSS version 27 [59] was used for all analyses.

## Results

### Sample

The study sample included 566 DMD caregivers and 594 comparison caregivers, representing nearly equally the four Child Age Group strata (see Additional file 5: Figure S1). The first three tables provide the univariate characteristics of the study samples; p-values from t-tests or chi-squared statistics assessing their differences; and explained variance (eta- or phi-squared) for those differences with *p* < 0.05. We dealt with the issue of multiple comparisons by focusing on explained variance only when *p* < 0.05 and in this way were selective about interpreting differences. The explained variance is mentioned parenthetically below. Whether due to DMD caregiving or other unmeasured factors, we found a number of small and medium effect-size differences between the two groups using Cohen’s criterion [60] (i.e., explaining 1.0–5.9% or 6.0–13.9% of the variance, respectively).

### Demographics

The DMD caregivers were younger than the comparison group (1% explained variance), more likely to be female (4%), more likely to be married or cohabiting and not divorced or widowed (1%), and less likely to be Hispanic (0.4%) (Table 1). The DMD caregivers reported fewer comorbidities (1%), including less frequent reports of back pain (1%), diabetes (3%), and high blood pressure (2%). While the two groups were similar in terms of body mass index, DMD caregivers were less likely to have smoked or vaped, whether recently or in the past (7%). DMD caregivers were less likely to be employed or retired and more likely to be unemployed (7%) and, among those who worked, were less likely to work 30 or more hours per week (4%).

**Table 1:** Descriptive Statistics of Caregivers

Variable	DMD caregivers (n = 566)				Comparison caregivers (n = 594)				*p* from *T*- or chi-square test	Variance explained If *p *< .05
	Mean	SD	Min	Max	Mean	SD	Min	Max		
Age	41.6	8.8	21	72	43.8	11.7	20	77	< 0.0005	0.01
Gender	**Frequency**	**%**			**Frequency**	**%**			< 0.0005	0.04
Male	140	25%			267	45%				
Female	426	75%			327	55%				
Marital status									0.04	0.01
Never married	31	5%			44	7%				
Married	453	80%			454	76%				
Cohabitation/domestic partner	40	7%			31	5%				
Separated	11	2%			9	2%				
Divorced	23	4%			45	8%				
Widowed	5	1%			10	2%				
Missing	3	1%			1	0%				
Race (check all that apply)										
Black	47	8%			69	12%			0.06	
White	519	92%			525	88%			0.05	
Other	21	4%			49	8%			NA	
Hispanic ethnicity									0.045	0.004
Yes	57	10%			83	14%				
No	490	87%			495	83%				
Missing	19	3%			16	3%				
Level of education									< 0.0005	0.11
Less than 12th grade	6	1%			2	0%				
High school diploma	59	10%			54	9%				
Technical (Vocational) degree	66	12%			15	3%				
Some college	92	16%			83	14%				
2-year University degree	89	16%			51	9%				
4-year University degree	171	30%			188	32%				
Masters degree	53	9%			183	31%				
Doctoral degree	5	1%			18	3%				
Missing	25	4%			0	0%				
Recruitment Source									NA	
Rare patient voice	49	9%			0	0%				
Patient advocacy groups	87	15%			0	0%				
Word of mouth	428	76%			0	0%				
IPSOS	0	0%			594	100%				
Missing	2	0%			0	0%				
	**Mean**	**SD**	**Min**	**Max**	**Mean**	**SD**	**Min**	**Max**		
Comorbidities, out of 15 presented	1.3	1.7	0	9	1.6	1.7	0	10	< 0.01	0.01
Specific Comorbidities*	**Frequency**	**%**			**Frequency**	**%**				
Arthritis	76	13%			85	15%			0.45	
Asthma	50	9%			67	12%			0.10	
Back Pain	189	33%			228	40%			0.02	0.01
Cancer now or in the past	18	3%			24	4%			0.35	
Depression	131	23%			130	23%			0.92	
Diabetes	20	4%			71	13%			< 0.0005	0.03
Heart Disease	11	2%			14	3%			0.50	
High Blood Pressure	53	9%			114	20%			< 0.0005	0.02
Insomnia	117	21%			98	17%			0.15	
Kidney Disease	3	1%			7	1%			0.23	
Liver Disease	5	1%			3	1%			0.44	
Lung Disease	3	1%			6	1%			0.35	
Stroke	3	1%			9	2%			0.10	
Ulcer or Stomach Disease	18	3%			20	4%			0.74	
Other	66	12%			55	10%			0.30	
	**Mean**	**SD**	**Min**	**Max**	**Mean**	**SD**	**Min**	**Max**		
BMI	26.9	6.0	16.5	40.0	27.1	5.8	15	40	0.49	
Smoking Status	**Frequency**	**%**			**Frequency**	**%**			< 0.0005	0.07
Never Smoked	447	79%			337	57%				
Used to Smoke	58	10%			68	11%				
Some Days Currently	23	4%			70	12%				
Every Day Currently	35	6%			116	20%				
Missing	3	1%			3	1%				
Work Status									< 0.0005	0.07
Employed	323	57%			432	73%				
Unemployed	202	36%			91	15%				
Retired	10	2%			40	7%				
Disabled due to medical condition	10	2%			20	3%				
Missing	21	4%			11	2%				
Hours Worked per Week									< 0.0005	0.04
Does not apply	242	43%			162	27%				
< 20	15	3%			12	2%				
20-29	41	7%			33	6%				
30-39	75	13%			139	23%				
40+	193	34%			248	42%				
COVID-Specific Variables										
Whether anyone in household infected (%)									< 0.0005	0.04
Definitely no	489	86%			440	74%				
Probably no	58	10%			79	13%				
Probably yes	8	1%			29	5%				
Definitely yes	3	1%			33	6%				
	**Mean**	**SD**	**Min**	**Max**	**Mean**	**SD**	**Min**	**Max**		
Financial hardship	18.8	9.0	6	42	21.4	7.2	6	42	< 0.0005	0.02
Isolation	14.1	6.5	4	28	14.3	5.8	4	28	0.68	
Social support	16	4.9	0	23	16.5	5.2	0	23	0.15	
Continuity of healthcare	2.6	0.7	0	3	2.5	0.8	0	3	0.09	

*A non-response was counted as the absence of the comorbidity in question

Regarding the differential impact of the COVID-19 pandemic, DMD caregivers’ households were less likely than the comparison group’s to have been infected with COVID-19 (explained variance 4%), and the former reported less financial hardship during the pandemic specifically (2%) (Table 1). There were no differences in terms of social support, sense of isolation, or continuity of healthcare.

### Adjusting for Demographic Differences via Propensity Scores

Given the above many demographic differences, the use of propensity scores helped control for variables that could have confounded observed group differences on outcomes. The propensity scores explained 11–15% of the variance in the data from the Cox & Snell and Nagelkerke pseudo-R^2^ statistics, respectively. The propensity scores were largely driven by Group differences in caregiver age, gender, race, and smoking/vaping status (see Additional file 1: Table S1 for details).

### Care recipient characteristics

Table 2 describes the care-recipient characteristics and, where relevant, a contrast of the DMD and comparison caregiver samples, again along with p-values from t-tests or chi-squared tests and explained variance for statistically significant differences. Care recipients for the comparison caregiver sample would be their own children. DMD caregivers reported providing support for one to five people with DMD (mean = 1.1, SD = 0.4), of whom up to three were their own children (Table 2). Ninety-seven percent of these caregivers were parents of the DMD index person (Table 2). An average of two people other than the caregiver were living in the household and providing support to the person(s) with DMD. The index DMD child had a mean age of 13.5 and an average of 1.6 comorbidities out of 15 presented, the most prevalent of which were anxiety, learning disabilities, attention deficit, sleep disorder, being overweight, scoliosis, and depression. The DMD care recipients were generally more likely to have each of the listed comorbidities than the comparison group with the exception of no differences on the prevalence of asthma and diabetes.

**Table 2:** Descriptive statistics of caregiving context

	DMD Care Recipients (n=566)				Comparison Care Recipient (n=594)				*p* from *T*- or Chi-Square Test	Variance explained If *p* < 0.05
Variable										
		%				%				
Index Child: % Male		100%				57%				
	Mean	SD	Min	Max	Mean	SD	Min	Max		
Age	13.5	6.7	2	42	14.1	10.9	0	55	0.22	
Years Cared for by This Caregiver	11.6	7.0	0	42	NA	NA	NA	NA	NA	
Number of People with DMD Caring for	1.1	0.4	1	5	NA	NA	NA	NA	NA	
Total Number of Children	1.9	1.0	0	8	1.9	1.1	0	8	0.94	
Number of Children with DMD	1.1	0.3	0	3	NA	NA	NA	NA	NA	
Number of Supports Living in the Home	2.1	0.8	0	3	2.0	0.8	0	3	0.03	0.004
Caregiver's Relationship to DMD Index Person	Frequency	%								
Parent	549	97%			NA	NA				
Sibling	3	1%			NA	NA				
Other Relative	9	2%			NA	NA				
Paid Caregiver	0	0%			NA	NA				
Other	5	1%			NA	NA				
	Mean	SD	Min	Max	Mean	SD	Min	Max		
Comorbidities, out of 11 presented	1.6	1.8	0	9	0.6	1.2	0	11	< 0.0005	0.09
Specific Comorbidities*	Frequency	%			Frequency	%				
Anxiety	204	36%			77	13%			< 0.0005	0.07
Asthma	45	8%			71	12%			0.06	
Attention Deficit	91	16%			36	6%			< 0.0005	0.02
Autism Spectrum Disorder	45	8%			12	2%			< 0.0005	0.02
Depression	74	13%			48	8%			0.004	0.01
Diabetes	11	2%			24	4%			0.29	
Epilepsy	17	3%			6	1%			0.008	0.01
Overweight	96	17%			48	8%			< 0.0005	0.02
Learning disabilities	130	23%			24	4%			< 0.0005	0.08
Scoliosis	85	15%			12	2%			< 0.0005	0.06
Sleep disorder	96	17%			30	5%			< 0.0005	0.04

*A non-response was counted as the absence of the comorbidity in question

### Unique aspects of DMD caregiving

#### Caregiver impact

**Fig. 1:**
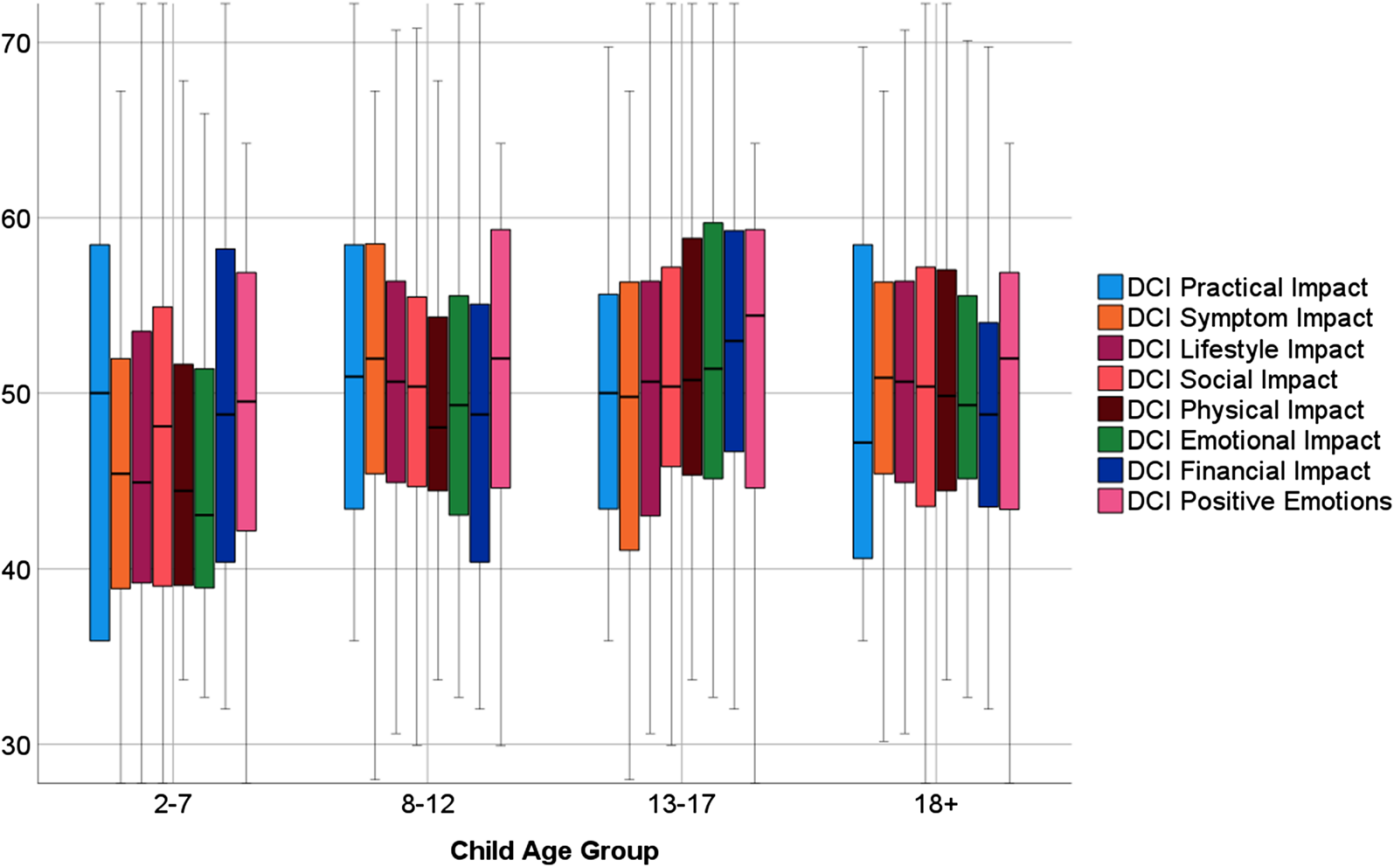
DMD Caregiver Impact (DCI) subscale scores by Child Age Group. These box-and-whiskers plots show the distributions of the eight DCI subscale scores by DMD Child Age Group strata. There were upward trends as the age of the index child increased through age 13–17, in all domains except Practical Impact and Positive Emotions

Figure 1 shows box-and-whiskers plots of the eight DCI subscales by DMD Child Age Group, and Additional file 3: Table S2 shows the descriptive statistics of the subscales. An analysis of variance model revealed statistically significant differences by Child Age Group in Emotional Impact (6% explained variance), Symptom and Physical Impact (5% in both), and Lifestyle, Social, and Financial Impact (each 3%) (Additional file 3: Table S2). We found upward trends as the age of the index child increased through age 13–17, in all domains except Practical Impact and Positive Emotions.

#### Home and vehicle accommodations

**Fig. 2:**
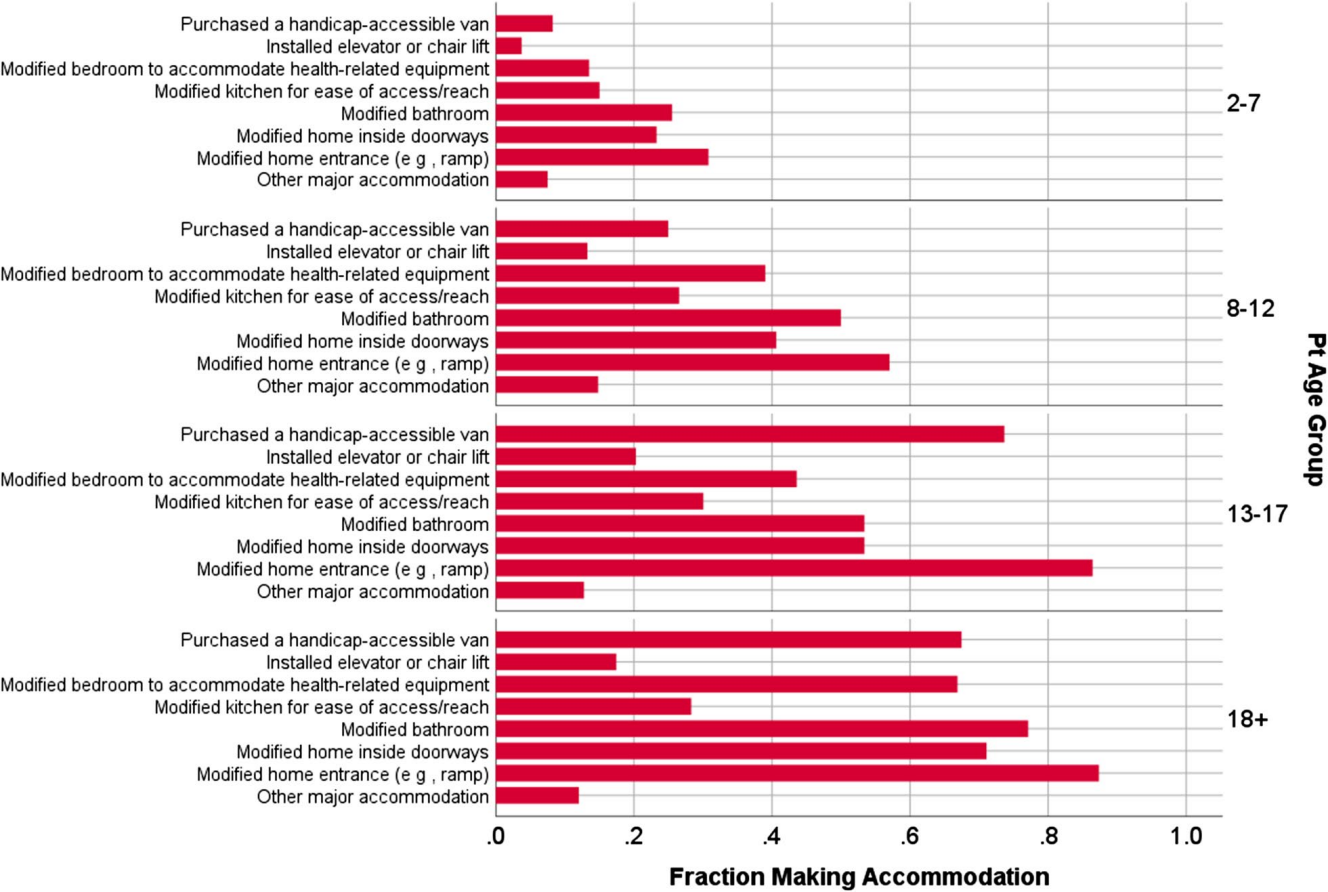
Home accommodations by child age status. DMD caregivers reported a greater frequency of home accommodations for older children

Table 3 provides information about the types of accommodations reported by DMD caregivers to make their home or vehicle more accessible for their child with DMD. The most prevalent were modifying their home entrance (67%), bathroom (53%), or inside-home doorways (49%); purchasing a handicap-accessible van (45%); and modifying a bedroom (43%). About a quarter of parents modified the kitchen and 14% installed an elevator. Figure 2 shows the greater frequency for home accommodations for older children.

**Table 3.** Descriptive statistics of outcomes measured

Construct	Measure	DMD caregiver				Comparison caregiver				*P* from *T*-test	Variance explained If *p* < .05
Generic QOL		Mean	SD	Min	Max	Mean	SD	Min	Max		
	PROMIS-10, Physical	50.3	10.2	20	68	48.3	8.0	27	68	< 0.0005	0.01
	PROMIS-10, Mental	49.0	10.1	21	68	50.3	8.0	25	68	0.02	0.01
	NeuroQOL Positive Affect and Well-Being	53.4	6.3	30	68	55.1	6.8	26	68	< 0.0005	0.02
	Ryff Environmental Mastery	30.1	6.6	13	42	30.9	6.8	11	42	0.045	0.003
	Resilience	0.0	1.0	-6	5	0.0	1.0	-7	5	0.78	
	Life Stress Questionnare	35.8	13.5	18	78	35.9	14.2	18	90	0.85	
	Difficulty Paying Bills	2.2	1.1	1	5	1.9	1.1	1	5	< 0.0005	0.02
Indirect Costs		**Mean**	**SD**	**Min**	**Max**						
	Count of accomodations	3.1	2.2	0	8	NA	NA	NA	NA	NA	NA
		**Frequency**	**%**								
	Modified home entrance	379	67%			NA	NA	NA	NA	NA	NA
	Modified bathroom	300	53%			NA	NA	NA	NA	NA	NA
	Modified inside home doorways	277	49%			NA	NA	NA	NA	NA	NA
	Handicap-accessible van	255	45%			NA	NA	NA	NA	NA	NA
	Modified bedroom	243	43%			NA	NA	NA	NA	NA	NA
	Modified kitchen	142	25%			NA	NA	NA	NA	NA	NA
	Installed elevator	79	14%			NA	NA	NA	NA	NA	NA
	Other	68	12%			NA	NA	NA	NA	NA	NA
		**Mean**	**SD**	**Min**	**Max**	**Mean**	**SD**	**Min**	**Max**		
	Hours Missed from Work	4.6	8.7	0	65	3.4	7.6	0	48	0.04	0.01
	Occupational Complexity	3.2	1.3	1	5	3.6	1.2	1	5	< 0.0005	0.03
	Impact on Siblings	34.8	12.4	9	62	NA	NA	NA	NA	NA	NA
	Had to help with DMD caregiving	5.1	1.7	1	7	NA	NA	NA	NA	NA	NA
	Had to give up time with friends	4.2	1.9	1	7	NA	NA	NA	NA	NA	NA
	Had to give up sports or other extracurricular activities	3.9	2.1	1	7	NA	NA	NA	NA	NA	NA
	Difficult to arrange their activities (e g , transportation)	3.9	1.7	1	7	NA	NA	NA	NA	NA	NA
	Had to give up summer camp or travel	3.9	2.1	1	7	NA	NA	NA	NA	NA	NA
	Didn't present them with opportunities because didn't want patient to suffer	3.4	2.0	1	7	NA	NA	NA	NA	NA	NA
	Had to select a college close to home	3.2	2.1	1	7	NA	NA	NA	NA	NA	NA
	Had to give up going to college	2.8	2.0	1	7	NA	NA	NA	NA	NA	NA
	Plenty of money available for their activities or schooling	4.1	1.9	1	7	NA	NA	NA	NA	NA	NA

#### Impact on siblings

Table 3 also provides information on the impact of DMD on the index child’s siblings. In addition to having to help with DMD caregiving, many siblings gave up time with friends (59% slightly, moderately, or strongly agreed), sports or extracurricular activities (52%), and/or summer camp or travel (44%). Also, a fair number of DMD caregivers (52%) reported that there was insufficient finances for siblings’ activities or schooling. On the other hand, participants tended to disagree that, due to DMD in the family, siblings had lost other opportunities, chosen only colleges close to home, or given up on going to college.

### Caregiver group differences on outcomes

Table 3 provides the descriptive statistics and unadjusted comparisons on outcomes. Table 4 and Additional file 3: Table S3 show results of the general linear models that assessed Group differences after adjusting for propensity score. The DMD Caregivers reported better physical health but worse mental health, positive affect/well-being, environmental mastery, and difficulty paying bills (explained variance 0.5%, 0.4%, 1.3%, 0.4%, and 2.5%, respectively; two of these met Cohen’s [60] criterion for “small” effect size; Table 3). There were no Group differences on resilience or life stress in these covariate-adjusted models.

**Table 4.** Group effects from general linear models adjusted for propensity scores

Dependent variable	b for DMD caregiver	Std. error	*t*	*p*	95% confidence interval		Partial Eta squared
					Lower bound	Upper bound	
PROMIS physical	1.45	0.58	2.47	0.01	0.30	2.60	0.005
PROMIS mental	− 1.26	0.58	− 2.17	0.03	− 2.41	− 0.12	0.004
NeuroQOL positive affect & well-being	− 1.61	0.42	− 3.86	0.00	− 2.43	− 0.79	0.013
Ryff environmental mastery	− 0.92	0.43	− 2.15	0.03	− 1.76	− 0.08	0.004
Resilience	− 0.10	0.06	− 1.54	0.12	− 0.22	0.03	0.002
Stress	1.14	0.88	1.29	0.20	− 0.59	2.87	0.001
Difficulty paying bills	0.39	0.07	5.41	0.00	0.25	0.53	0.025
Hours missed from work*	1.68	0.63	2.68	0.01	0.45	2.91	0.009

*Excludes those not working

A separate set of models examined the differential impact of Child Age Group on outcomes via interactions between caregiver group (“Group”) and Child Age Group. These models showed that, compared to controls and after adjusting for propensity scores, DMD caregivers reported worse physical health, mental health, positive affect, environmental mastery, stressful life events, and difficulty paying bills than comparison caregivers. DMD caregivers reported worse outcomes if their DMD child was in certain age groups, with the outcomes and corresponding age groups as follows: physical health (age 13+); mental health (age 13–17); positive affect (age 18+); environmental mastery (age 8+); stress (age 13+); and difficulty paying bills (age 13+) (Table 5 and Additional file 4: Table S4).

**Table 5.** Group and Child Age Main- and Interaction-Effects from General Linear Models Adjusted for Propensity Scores

Dependent variable	Parameter	b	Std. error	*t*	*p*	95% Confidence interval		Partial eta squared
						Lower bound	Upper bound	
PROMIS physical	Child Age 18+	0.33	1.07	0.31	0.76	− 1.78	2.43	0.000
	Child Age 13–17	1.20	1.09	1.10	0.27	− 0.94	3.34	0.001
	Child Age 8–12	− 0.89	1.00	− 0.89	0.37	− 2.85	1.06	0.001
	DMD Caregiver	6.00	1.07	5.60	0.00	3.90	8.10	0.027
	DMD Caregiver * Child Age 18+	− 6.45	1.48	− 4.34	0.00	− 9.36	− 3.53	0.017
	DMD Caregiver * Child Age 13–17	− 9.00	1.55	− 5.81	0.00	− 12.04	− 5.96	0.029
	DMD Caregiver * Child Age 8–12	− 2.08	1.49	− 1.40	0.16	− 4.99	0.84	0.002
PROMIS mental	Child Age 18+	0.49	1.08	0.45	0.65	− 1.63	2.61	0.000
	Child Age 13–17	2.76	1.10	2.51	0.01	0.60	4.92	0.006
	Child Age 8–12	0.64	1.01	0.63	0.53	− 1.34	2.61	0.000
	DMD Caregiver	3.14	1.08	2.90	0.00	1.02	5.26	0.008
	DMD Caregiver * Child Age 18+	− 4.49	1.50	− 3.00	0.00	− 7.43	− 1.55	0.008
	DMD Caregiver * Child Age 13–17	− 8.99	1.56	− 5.76	0.00	− 12.05	− 5.93	0.029
	DMD Caregiver * Child Age 8–12	− 4.22	1.50	− 2.81	0.00	− 7.16	− 1.28	0.007
NeuroQOL positive affect and well being	Child Age 18+	0.50	0.78	0.64	0.52	− 1.03	2.04	0.000
	Child age 13–17	0.65	0.80	0.82	0.41	− 0.91	2.22	0.001
	Child age 8–12	0.76	0.73	1.04	0.30	− 0.67	2.19	0.001
	DMD caregiver	− 0.49	0.78	− 0.63	0.53	− 2.03	1.04	0.000
	DMD caregiver * Child age 18+	− 2.41	1.08	− 2.22	0.03	− 4.54	− 0.28	0.004
	DMD caregiver * Child age 13–17	− 1.46	1.13	− 1.29	0.20	− 3.68	0.76	0.001
	DMD caregiver * Child age 8–12	− 0.15	1.09	− 0.14	0.89	− 2.28	1.98	0.000
Ryff environmental mastery	Child age 18+	2.79	0.79	3.53	0.00	1.24	4.34	0.011
	Child age 13–17	1.89	0.81	2.34	0.02	0.30	3.47	0.005
	Child age 8–12	0.85	0.74	1.15	0.25	− 0.60	2.29	0.001
	DMD caregiver	2.68	0.79	3.38	0.00	1.13	4.23	0.010
	DMD caregiver * Child age 18+	− 5.46	1.10	− 4.98	0.00	− 7.62	− 3.31	0.022
	DMD caregiver * Child age 13–17	− 6.11	1.14	− 5.34	0.00	− 8.36	− 3.87	0.025
	DMD caregiver * Child age 8–12	− 3.36	1.10	− 3.06	0.00	− 5.51	− 1.21	0.008
Resilience	Child age 18+	0.01	0.12	0.07	0.94	− 0.22	0.24	0.000
	Child age 13–17	0.07	0.12	0.57	0.57	− 0.17	0.31	0.000
	Child Age 8–12	0.03	0.11	0.25	0.80	− 0.19	0.24	0.000
	DMD Caregiver	− 0.07	0.12	− 0.57	0.57	− 0.30	0.17	0.000
	DMD Caregiver * Child Age 18+	0.11	0.16	0.66	0.51	− 0.21	0.43	0.000
	DMD Caregiver * Child Age 13–17	− 0.30	0.17	− 1.75	0.08	− 0.64	0.04	0.003
	DMD Caregiver * Child Age 8–12	0.00	0.16	0.00	1.00	− 0.32	0.32	0.000
Stress	Child Age 18+	− 7.51	1.61	− 4.65	0.00	− 10.68	− 4.35	0.019
	Child Age 13–17	− 5.64	1.64	− 3.43	0.00	− 8.86	− 2.41	0.010
	Child Age 8–12	− 1.16	1.50	− 0.77	0.44	− 4.11	1.79	0.001
	DMD Caregiver	− 5.70	1.61	− 3.53	0.00	− 8.87	− 2.53	0.011
	DMD Caregiver * Child Age 18+	10.58	2.24	4.73	0.00	6.19	14.96	0.020
	DMD Caregiver * Child Age 13–17	15.60	2.33	6.69	0.00	11.02	20.17	0.039
	DMD Caregiver * Child Age 8–12	3.90	2.24	1.74	0.08	− 0.50	8.29	0.003
Difficulty Paying Bills	Child Age 18+	− 0.40	0.13	− 3.00	0.00	− 0.66	− 0.14	0.008
	Child Age 13–17	− 0.30	0.14	− 2.17	0.03	− 0.56	− 0.03	0.004
	Child Age 8–12	− 0.06	0.12	− 0.49	0.63	− 0.30	0.18	0.000
	DMD Caregiver	0.02	0.13	0.11	0.91	− 0.25	0.28	0.000
	DMD Caregiver * Child Age 18+	0.53	0.19	2.84	0.00	0.16	0.89	0.007
	DMD Caregiver * Child Age 13–17	0.88	0.19	4.56	0.00	0.50	1.26	0.018
	DMD Caregiver * Child Age 8–12	0.26	0.19	1.39	0.17	− 0.11	0.62	0.002
Hours Missed from Work*	Child Age 18+	− 2.16	1.20	− 1.80	0.07	− 4.51	0.19	0.004
	Child Age 13–17	− 1.03	1.09	− 0.95	0.34	− 3.17	1.10	0.001
	Child Age 8–12	− 0.42	1.00	− 0.42	0.68	− 2.38	1.55	0.000
	DMD Caregiver	3.28	1.13	2.90	0.00	1.06	5.50	0.011
	DMD Caregiver * Child Age 18+	− 0.77	1.65	− 0.47	0.64	− 4.01	2.47	0.000
	DMD Caregiver * Child Age 13–17	− 2.37	1.76	− 1.35	0.18	− 5.83	1.08	0.002
	DMD Caregiver * Child Age 8–12	− 2.25	1.62	− 1.39	0.17	− 5.43	0.93	0.003

*Excludes those not working

### Caregiver group differences on work productivity

A general linear model adjusting for propensity score suggested that employed DMD caregivers missed more hours from work than comparison caregivers (explained variance 0.9%; Table 4). A separate model revealed no differential effect by Child Age Group (Table 5).

### Caregiver group differences on unrealized ambitions

**Fig. 3:**
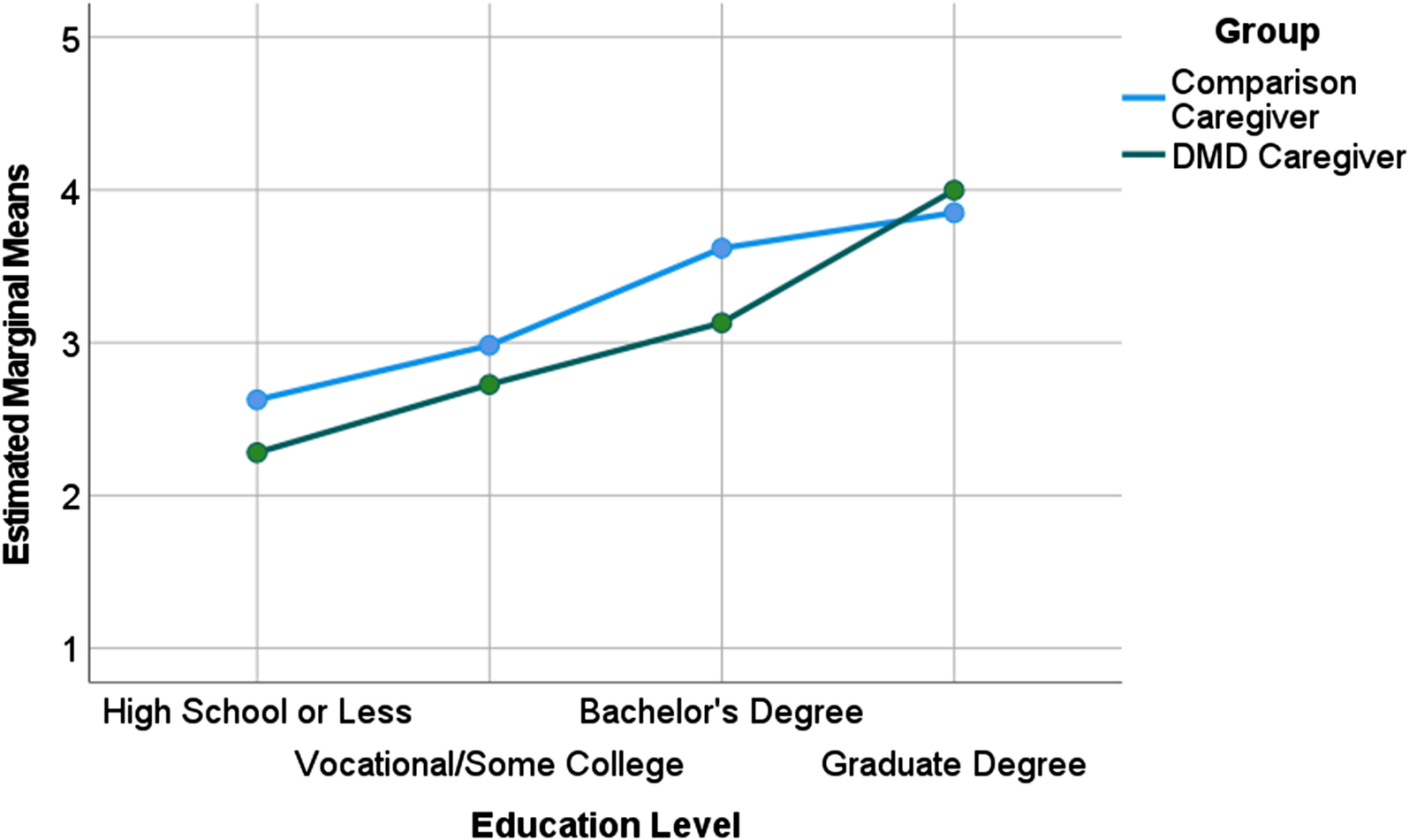
Occupational Complexity achieved by DMD versus comparison caregivers. DMD caregivers showed lower levels of occupational complexity after adjusting for education, across all levels with the exception of graduate-level training

DMD parents were much more likely than comparison parents to have a technical degree, some college, or a 2-year college degree (44% vs. 26% of the samples, respectively). DMD caregivers were less likely to have a graduate-level education (10% versus 34% of the samples respectively; Table 1). Explained variance for education was high at 11%. Moreover, of the two groups, DMD caregivers showed levels of occupational complexity that, after adjusting for propensity score and education, were on average 0.15 points lower on a 1–5 scale. This was essentially true at all levels except graduate-level training (Fig. 3). These findings were similar in models including and excluding caregivers without work history. In the latter model, missing values of O*NET were imputed as “2”, reflecting being a caregiver, for 139 and 56 in DMD caregiver and comparison groups, respectively. There was no link between Child Age Group and occupational complexity. Further, of the two groups, the DMD caregivers under-achieved with respect to community education norms, as measured by ZIP-code educational averages (0.8% explained variance), after controlling for propensity score.

## Discussion

The present work revealed that DMD caregivers appear to fare worse on most outcomes than a nationally representative comparison group, even after adjusting for possible confounders. Upon further inspection, it became clear that providing caregiving support for DMD teenage children was the most challenging. This is a difficult age for any parent, but boys with DMD are also becoming increasingly dependent upon a wheelchair for mobility, giving rise to increased intensity of care and physical demands on caregivers. While DMD caregivers seemed to have better physical health in simpler models, more complex models that considered the child’s age group showed a greater toll taken on DMD caregivers of adult and especially teenage children. We found no Group difference in main effects of resilience or stress, but further analysis revealed a greater strain for the latter outcome when caring for children with DMD who were teenage or older (interactions). The policy implications of these findings are that considering the caregiver impact would be highly relevant to determining the value and cost-effectiveness of DMD therapies. Effective treatment is associated with lower caregiver impact in advanced Parkinson’s disease, for example [61]. In addition to QOL challenges, DMD caregivers seemed to have restricted their professional ambitions. They were less likely than the comparison caregivers to work or work full-time, and more likely to miss time from work. They were much less likely to complete a college degree and less likely to achieve educationally what would be expected from community norms. Even among those with a college education, DMD caregivers achieved less complex occupations than controls. Further, many DMD caregivers reported that their other children had given up time with friends, sports, extracurricular activities, and/or summer camp or travel due to the index child’s DMD. These differences suggest constraints on life choices, and presence of hidden restrictions due to DMD caregiving. All of these hidden and not-so-hidden restrictions could be mitigated by effective treatment. Future research might, for example, investigate whether a DMD treatment is associated with better QOL outcomes in the short term, and higher educational or occupational achievement in the long term.

The comparability in resilience between the two groups is noteworthy given the extensive difficulties faced by DMD caregivers. In addition to the many costly accommodations they had to make as the child’s DMD progressed, caregivers reported a general worsening on many aspects of the DCI. Nonetheless, DMD caregivers’ DCI Positive Emotions scores were consistent across child age groups despite older children’s greater caregiving impact indicated by the other domain scores. Managing to maintain engagement and positivity in the face of DMD adversities speaks to an inner strength and grit worthy of further investigation. It may also signal response-shift effects [62], referring to changes in internal standards, values and/or conceptualization of quality of life, all of which can also be investigated in future longitudinal research.

Resilience in maintaining responsibilities in the face of DMD caregiving may relate to better self-care. DMD caregivers were less likely to smoke or vape than comparison caregivers (never smoked: 79% vs. 57%), and had avoided COVID-19 infection better than the comparison group had (probably or definitely no infection: 96% vs. 87%). DMD Caregivers also suffered less financial hardship than comparison caregivers, which could be partially due to a greater proportion of them being unemployed to begin with, so Covid-19 did not appreciably change their financial circumstances. This finding is consistent with past research that documented lower perceived impact among those caregivers who practice more health-promoting behaviors [63]. Future research might address whether DMD caregivers are more likely to engage in reserve-building activities that stimulate the brain and lead to greater flexibility and more adaptive ways of coping [64–69].

The present study has distinct advantages over past research cited in our Introduction in its ample sample size, inclusion of a nationally representative comparison group, coverage of age-related subgroups, and measurement of diverse yet relevant constructs. The majority of prior literature has not characterized the demands placed upon parent-caregivers over time. The present study described the trajectory of caregiving over time and across different stages of the disease, likely reflecting the increasing complexity and scope of caregiver responsibilities as people with DMD progress through the disease.

The limitations of the present work must, however, be acknowledged. Because of the snowball sampling employed, it is not possible to calculate a response rate or to assess selection biases. Second and similar to much caregiver research, the DMD caregivers are disproportionately female, whereas the comparison group is more balanced. Third, the study sample does not include enough Hispanic, Black, Asian-American, or Native American participants to enable subgroup analyses. Future research might address caregiver impact in these important cohorts. Fourth, in order to include all caregivers in the occupational complexity analysis, we replaced each missing value with a “2”, reflecting published benchmarks for “caregiver.” This imputation yielded greater power but, as it was applied disproportionately to DMD caregivers, it may have led to an underestimate of their occupational complexity. Fifth, educational differences between the caregiver samples may have pre-dated the arrival of their children, so it is unclear whether these differences are a direct effect of differences in caregiving demands. Sixth, this case-control study has limitations due to its quasi-experimental design. One cannot randomize individuals to be caregivers of people with DMD, so causal inference is difficult. Nonetheless, the inclusion of a general-population comparison cohort facilitates some confidence in the study’s conclusions. Seventh, the mean age of the DMD cohort was 13.5 years, so it is possible that the findings are skewed to an older cohort. Those with younger children (i.e., who were more recently diagnosed) may have differing impacts. Nonetheless, this study serves to highlight that even after many years of diagnosis with DMD, parent-caregivers do continue to struggle, perhaps as a product of the progressive nature of the illness. Finally, the outcome differences noted, including those related to child age, are based on cross-sectional data. A longitudinal assessment could address whether such differences persist within families.

In summary, DMD has a high and broad impact, not only on the patients who suffer from this disease but also on their caregivers. DMD caregivers fared worse on most QOL outcomes and faced more hurdles in their educational and work life. The severity of DMD was associated with caregiver impact, and caring for teenagers with DMD was particularly challenging have greater impact. Findings from this study suggest that DMD caregivers face numerous constraints and indirect costs that impact their health, well-being, and financial welfare. Nonetheless, parents of DMD children of all ages maintained notable resilience and positivity.

## Supplementary Information

**Additional file 1. Supplemental Table 1.** Propensity Score Model.

**Additional file 2. Supplemental Table 2.** DMD Caregiver Impact Descriptive Statistics and ANOVA results.

**Additional file 3. Supplemental Table 3.** Full Output of General Linear Models Evaluating Group Effects adjusted for propensity scores.

**Additional file 4. Supplemental Table 4.** Group and Child Age Main- and Interaction-Effects from General Linear Models Adjusted for Propensity Scores.

**Additional file 5. Supplemental Figure 1.** Bar chart showing numbers of DMD and comparison caregivers in the four Child Age Group strata.

## Data Availability

The study data are confidential and thus not able to be shared.

## Abbreviations

DMD: Duchenne muscular dystrophy
LLC: Limited liability corporation
HRQOL: Health-related quality of life
QOL: Quality of life
