# Supplementary material for: Characterizing the quality-of-life impact of Duchenne muscular dystrophy on caregivers: a case-control investigation
Source: J Patient Rep Outcomes. 2021 Nov 20;5:124. doi: 10.1186/s41687-021-00386-y (PMC8605451; doi:10.1186/s41687-021-00386-y)
Supplement: Supplementary file 1 — Additional file 1. Supplemental Table 1. Propensity Score Model. [file 41687_2021_386_MOESM1_ESM.pdf]

Supplemental Table 1. Propensity Score Model

	B	S.E.	Wald	df	Sig.	Exp(B)
Year of birth	0.02	0.01	10.42	1.00	0.00	1.02
Gender	0.67	0.14	23.65	1.00	0.00	1.96
White Race	0.47	0.17	7.72	1.00	0.01	1.60
Marital status	-0.03	0.07	0.14	1.00	0.70	0.97
No. supports in the home	0.11	0.08	1.95	1.00	0.16	1.12
Smoke or vape	-0.46	0.07	50.10	1.00	0.00	0.63
No. children	0.00	0.06	0.00	1.00	0.97	1.00
Constant	-43.02	12.93	11.08	1.00	0.00	0.00
