# Supplementary material for: Characterizing the quality-of-life impact of Duchenne muscular dystrophy on caregivers: a case-control investigation
Source: J Patient Rep Outcomes. 2021 Nov 20;5:124. doi: 10.1186/s41687-021-00386-y (PMC8605451; doi:10.1186/s41687-021-00386-y)
Supplement: Supplementary file 2 — Additional file 2. Supplemental Table 2. DMD Caregiver Impact Descriptive Statistics and ANOVA results. [file 41687_2021_386_MOESM2_ESM.pdf]

| Supplemental Table 2. DMD Caregiver Impact Descriptive Statistics and ANOVA results | | | | | | | |
|---|---|---|---|---|---|---|---|
| | | | | | | Age-Group Differences | |
| DMD Caregiver Impact (DCI) Subscale | Mean | SD | Min | Max | Cronbach's Alpha | *P* from *ANOVA* | Variance Explained If *P* <.05 |
| Practical Impact | 50 | 10 | 36 | 81 | 0.88 | 0.243 | |
| Symptom Impact | 50 | 10 | 24 | 67 | 0.88 | <0.0005 | 0.05 |
| Lifestyle Impact | 50 | 10 | 28 | 74 | 0.77 | 0.001 | 0.03 |
| Social Impact | 50 | 10 | 28 | 73 | 0.87 | 0.001 | 0.03 |
| Physical Impact | 50 | 10 | 34 | 77 | 0.89 | <0.0005 | 0.05 |
| Emotional Impact | 50 | 10 | 33 | 74 | 0.90 | <0.0005 | 0.06 |
| Financial Impact | 50 | 10 | 32 | 74 | 0.88 | 0.003 | 0.03 |
| Positive Emotions | 50 | 10 | 15 | 64 | 0.85 | 0.137 | |
