# Supplementary material for: Characterizing the quality-of-life impact of Duchenne muscular dystrophy on caregivers: a case-control investigation
Source: J Patient Rep Outcomes. 2021 Nov 20;5:124. doi: 10.1186/s41687-021-00386-y (PMC8605451; doi:10.1186/s41687-021-00386-y)
Supplement: Supplementary file 3 — Additional file 3. Supplemental Table 3. Full Output of General Linear Models Evaluating Group Effects adjusted for propensity scores. [file 41687_2021_386_MOESM3_ESM.pdf]

Supplemental Table 3. Full Output of General Linear Models Evaluating Group Effects adjusted for propensity scores

Tests of Between-Subjects Effects

Source		Type III Sum of Squares	df	Mean Square	F	Sig.	Partial Eta Squared
**Corrected Model**	PROMIS Physical	1619.740[a]	2	809.87	9.73	0.000	0.02
	PROMIS Mental	522.810[b]	2	261.41	3.15	0.043	0.01
	NeuroQOL Pos.Affect	772.008[c]	2	386.00	9.09	0.000	0.02
	Ryff Envr. Mastery	233.258[d]	2	116.63	2.62	0.074	0.00
	Resilience	10.860[e]	2	5.43	5.58	0.004	0.01
	Stress	2167.493[f]	2	1083.75	5.71	0.003	0.01
	Difficulty paying bills	39.229[g]	2	19.61	15.55	0.000	0.03
**Intercept**	PROMIS Physical	245444.41	1	245444.41	2949.12	0.000	0.72
	PROMIS Mental	274828.82	1	274828.82	3315.36	0.000	0.75
	NeuroQOL Pos.Affect	325812.50	1	325812.50	7671.34	0.000	0.87
	Ryff Envr. Mastery	96294.02	1	96294.02	2159.60	0.000	0.66
	Resilience	8.92	1	8.92	9.17	0.003	0.01
	Stress	175915.65	1	175915.65	927.57	0.000	0.45
	Difficulty paying bills	597.97	1	597.97	473.97	0.000	0.30
**Group**	PROMIS Physical	509.78	1	509.78	6.13	0.013	0.01
	PROMIS Mental	388.87	1	388.87	4.69	0.031	0.00
	NeuroQOL Pos.Affect	633.74	1	633.74	14.92	0.000	0.01
	Ryff Envr. Mastery	206.81	1	206.81	4.64	0.031	0.00
	Resilience	2.32	1	2.32	2.38	0.123	0.00
	Stress	315.02	1	315.02	1.66	0.198	0.00
	Difficulty paying bills	36.92	1	36.92	29.27	0.000	0.03
**Propensity Score**	PROMIS Physical	520.27	1	520.27	6.25	0.013	0.01
	PROMIS Mental	12.96	1	12.96	0.16	0.693	0.00
	NeuroQOL Pos.Affect	3.19	1	3.19	0.08	0.784	0.00
	Ryff Envr. Mastery	100.50	1	100.50	2.25	0.134	0.00
	Resilience	10.73	1	10.73	11.03	0.001	0.01
	Stress	2166.75	1	2166.75	11.42	0.001	0.01
	Difficulty paying bills	13.15	1	13.15	10.42	0.001	0.01
**Error**	PROMIS Physical	93130.34	1119	83.23			
	PROMIS Mental	92760.29	1119	82.90			
	NeuroQOL Pos.Affect	47525.48	1119	42.47			
	Ryff Envr. Mastery	49895.00	1119	44.59			
	Resilience	1089.50	1119	0.97			
	Stress	212220.70	1119	189.65			
	Difficulty paying bills	1411.77	1119	1.26			
**Total**	PROMIS Physical	2822321.91	1122				
	PROMIS Mental	2864396.07	1122				

	NeuroQOL Pos.Affect	3357230.93	1122
	Ryff Envr. Mastery	1095044.36	1122
	Resilience	1100.53	1122
	Stress	1651754.11	1122
	Difficulty paying bills	5943.00	1122
**Corrected Total**	PROMIS Physical	94750.08	1121
	PROMIS Mental	93283.10	1121
	NeuroQOL Pos.Affect	48297.49	1121
	Ryff Envr. Mastery	50128.26	1121
	Resilience	1100.36	1121
	Stress	214388.20	1121
	Difficulty paying bills	1451.00	1121

a. R Squared = .017 (Adjusted R Squared = .015)
b. R Squared = .006 (Adjusted R Squared = .004)
c. R Squared = .016 (Adjusted R Squared = .014)
d. R Squared = .005 (Adjusted R Squared = .003)
e. R Squared = .010 (Adjusted R Squared = .008)
f. R Squared = .010 (Adjusted R Squared = .008)
g. R Squared = .027 (Adjusted R Squared = .025)

Parameter Estimates

Dependent Variable		B	Std. Error	t	Sig.	95% Confidence Lower Bound	95% Confidence Upper Bound	Partial Eta Squared
**PROMIS Physical**	Intercept	46.54	0.81	57.12	0.000	44.94	48.13	0.745
	DMD Caregiver Group	1.45	0.58	2.47	0.013	0.30	2.60	0.005
	Comparison Caregiver Group	0[a]						
	Propensity Score	4.27	1.71	2.50	0.013	0.92	7.62	0.006
**PROMIS Mental**	Intercept	50.64	0.81	62.29	0.000	49.05	52.24	0.776
	DMD Caregiver Group	-1.26	0.58	-2.17	0.031	-2.41	-0.12	0.004
	Comparison Caregiver Group	0[a]						
	Propensity Score	-0.67	1.70	-0.40	0.693	-4.02	2.67	0.000
**NeuroQOL Pos.Affect**	Intercept	55.26	0.58	94.95	0.000	54.12	56.40	0.890
	DMD Caregiver Group	-1.61	0.42	-3.86	0.000	-2.43	-0.79	0.013
	Comparison Caregiver Group	0[a]						
	Propensity Score	-0.33	1.22	-0.27	0.784	-2.73	2.06	0.000
**Ryff Envr. Mastery**	Intercept	30.06	0.60	50.42	0.000	28.89	31.23	0.694
	DMD Caregiver Group	-0.92	0.43	-2.15	0.031	-1.76	-0.08	0.004
	Comparison Caregiver Group	0[a]						
	Propensity Score	1.88	1.25	1.50	0.134	-0.58	4.33	0.002

**Resilience**	Intercept	-0.24	0.09	-2.68	0.007	-0.41	-0.06	0.006
	DMD Caregiver Group	-0.10	0.06	-1.54	0.123	-0.22	0.03	0.002
	Comparison Caregiver Group	0[a]						
	Propensity Score	0.61	0.18	3.32	0.001	0.25	0.98	0.010
**Stress**	Intercept	39.44	1.23	32.07	0.000	37.03	41.85	0.479
	DMD Caregiver Group	1.14	0.88	1.29	0.198	-0.59	2.87	0.001
	Comparison Caregiver Group	0[a]						
	Propensity Score	-8.72	2.58	-3.38	0.001	-13.77	-3.66	0.010
**Difficulty paying bills**	Intercept	2.14	0.10	21.31	0.000	1.94	2.33	0.289
	DMD Caregiver Group	0.39	0.07	5.41	0.000	0.25	0.53	0.025
	Comparison Caregiver Group	0[a]						
	Propensity Score	-0.68	0.21	-3.23	0.001	-1.09	-0.27	0.009

a. This parameter is set to zero because it is redundant.

Tests of Between-Subjects Effects

Dependent Variable: Hours missed from work

Source	Type III Sum of Squares	df	Mean Square	F	Sig.	Partial Eta Squared
**Corrected Model**	600.392[a]	2	300.20	4.64	0.010	0.012
**Intercept**	2731.58	1	2731.58	42.27	0.000	0.053
**Propensity Score**	328.68	1	328.68	5.09	0.024	0.007
**Group**	463.64	1	463.64	7.17	0.008	0.009
**Error**	48600.34	752	64.63			
**Total**	60597.09	755				
**Corrected Total**	49200.73	754				

a. R Squared = .012 (Adjusted R Squared = .010)

Parameter Estimates

Dependent Variable: Hours missed from work

Parameter	B	Std. Error	t	Sig.	95% Confidence Lower Bound	95% Confidence Upper Bound	Partial Eta Squared
**Intercept**	5.07	0.85	5.97	0.000	3.40	6.74	0.045
**Propensity Score**	-4.12	1.83	-2.26	0.024	-7.71	-0.53	0.007
**DMD Caregiver Group**	1.68	0.63	2.68	0.008	0.45	2.91	0.009
**Comparison Caregiver Group**	0[a]						

a. This parameter is set to zero because it is redundant.
