# Supplementary material for: Characterizing the quality-of-life impact of Duchenne muscular dystrophy on caregivers: a case-control investigation
Source: J Patient Rep Outcomes. 2021 Nov 20;5:124. doi: 10.1186/s41687-021-00386-y (PMC8605451; doi:10.1186/s41687-021-00386-y)
Supplement: Supplementary file 4 — Additional file 4. Supplemental Table 4. Group and Child Age Main- and Interaction-Effects from General Linear Models Adjusted for Propensity Scores. [file 41687_2021_386_MOESM4_ESM.pdf]

Supplemental Table 4. Group and Child Age Main- and Interaction-Effects from General Linear Models Adjusted for Propensity Scores

Tests of Between-Subjects Effects

Source		Type III Sum of Squares	df	Mean Square	F	Sig.	Partial Eta Squared
**Corrected Model**	PROMIS Physical	6633.980[a]	8	829.25	10.47	0.000	0.070
	PROMIS Mental	3686.483[b]	8	460.81	5.72	0.000	0.040
	NeuroQOL Pos.Affect	1339.170[c]	8	167.40	3.97	0.000	0.028
	Ryff Envr. Mastery	2054.050[d]	8	256.76	5.94	0.000	0.041
	Resilience	20.207[e]	8	2.53	2.60	0.008	0.018
	Stress	14486.456[f]	8	1810.81	10.08	0.000	0.068
	Difficulty paying bills	79.327[g]	8	9.92	8.05	0.000	0.055
**Intercept**	PROMIS Physical	235973.80	1	235973.80	2980.60	0.000	0.728
	PROMIS Mental	259383.09	1	259383.09	3222.15	0.000	0.743
	NeuroQOL Pos.Affect	306569.73	1	306569.73	7266.28	0.000	0.867
	Ryff Envr. Mastery	89286.66	1	89286.66	2067.14	0.000	0.650
	Resilience	10.33	1	10.33	10.64	0.001	0.009
	Stress	171559.15	1	171559.15	955.20	0.000	0.462
	Difficulty paying bills	585.63	1	585.63	475.19	0.000	0.299
**Propensity Score**	PROMIS Physical	242.52	1	242.52	3.06	0.080	0.003
	PROMIS Mental	32.53	1	32.53	0.40	0.525	0.000
	NeuroQOL Pos.Affect	12.43	1	12.43	0.29	0.587	0.000
	Ryff Envr. Mastery	129.04	1	129.04	2.99	0.084	0.003
	Resilience	12.01	1	12.01	12.37	0.000	0.011
	Stress	3114.24	1	3114.24	17.34	0.000	0.015
	Difficulty paying bills	17.29	1	17.29	14.03	0.000	0.012
**Patient Age Category**	PROMIS Physical	1700.74	3	566.91	7.16	0.000	0.019
	PROMIS Mental	559.38	3	186.46	2.32	0.074	0.006
	NeuroQOL Pos.Affect	275.91	3	91.97	2.18	0.089	0.006
	Ryff Envr. Mastery	302.68	3	100.89	2.34	0.072	0.006
	Resilience	2.93	3	0.98	1.01	0.388	0.003
	Stress	2723.11	3	907.70	5.05	0.002	0.013
	Difficulty paying bills	11.81	3	3.94	3.19	0.023	0.009
**Group**	PROMIS Physical	613.68	1	613.68	7.75	0.005	0.007
	PROMIS Mental	387.40	1	387.40	4.81	0.028	0.004
	NeuroQOL Pos.Affect	523.30	1	523.30	12.40	0.000	0.011
	Ryff Envr. Mastery	260.92	1	260.92	6.04	0.014	0.005

Resilience	3.11	1	3.11	3.21	0.074	0.003
Stress	772.34	1	772.34	4.30	0.038	0.004
Difficulty paying bills	43.49	1	43.49	35.29	0.000	0.031

**Group * Patient Age Category**	PROMIS Physical	3365.57	3	1121.86	14.17	0.000	0.037
	PROMIS Mental	2675.23	3	891.74	11.08	0.000	0.029
	NeuroQOL Pos.Affect	280.44	3	93.48	2.22	0.085	0.006
	Ryff Envr. Mastery	1566.65	3	522.22	12.09	0.000	0.032
	Resilience	5.97	3	1.99	2.05	0.105	0.005
	Stress	9648.30	3	3216.10	17.91	0.000	0.046
	Difficulty paying bills	28.27	3	9.42	7.65	0.000	0.020
**Error**	PROMIS Physical	88116.10	1113	79.17			
	PROMIS Mental	89596.62	1113	80.50			
	NeuroQOL Pos.Affect	46958.32	1113	42.19			
	Ryff Envr. Mastery	48074.21	1113	43.19			
	Resilience	1080.16	1113	0.97			
	Stress	199901.74	1113	179.61			
	Difficulty paying bills	1371.67	1113	1.23			
**Total**	PROMIS Physical	2822321.91	1122				
	PROMIS Mental	2864396.07	1122				
	NeuroQOL Pos.Affect	3357230.93	1122				
	Ryff Envr. Mastery	1095044.36	1122				
	Resilience	1100.53	1122				
	Stress	1651754.11	1122				
	Difficulty paying bills	5943.00	1122				
**Corrected Total**	PROMIS Physical	94750.08	1121				
	PROMIS Mental	93283.10	1121				
	NeuroQOL Pos.Affect	48297.49	1121				
	Ryff Envr. Mastery	50128.26	1121				
	Resilience	1100.36	1121				
	Stress	214388.20	1121				
	Difficulty paying bills	1451.00	1121				

a. R Squared = .070 (Adjusted R Squared = .063)
b. R Squared = .040 (Adjusted R Squared = .033)
c. R Squared = .028 (Adjusted R Squared = .021)
d. R Squared = .041 (Adjusted R Squared = .034)
e. R Squared = .018 (Adjusted R Squared = .011)
f. R Squared = .068 (Adjusted R Squared = .061)
g. R Squared = .055 (Adjusted R Squared = .048)

Parameter Estimates

Dependent Variable		B	Std. Error	t	Sig.	95% Confidence Interval Lower Bound	Upper Bound	Partial Eta
**PROMIS Physical**	Intercept	46.992	1.102	42.639	0.000	44.830	49.155	0.620
	Propensity Score	3.028	1.730	1.750	0.080	-0.367	6.422	0.003
	Child Age 18+	0.327	1.072	0.305	0.760	-1.776	2.430	0.000
	Child Age 13-17	1.201	1.092	1.100	0.272	-0.942	3.343	0.001
	Child Age 8-12	-0.893	0.998	-0.895	0.371	-2.850	1.065	0.001
	Child Age 2-7	0[a]						
	DMD Caregiver Group	6.000	1.072	5.599	0.000	3.897	8.102	0.027
	Comparison Caregiver Group	0[a]						
	DMD Caregiver Group * Child Age 18+	-6.447	1.484	-4.343	0.000	-9.360	-3.535	0.017
	DMD Caregiver Group * Child Age 13-17	-8.998	1.548	-5.811	0.000	-12.035	-5.960	0.029
	DMD Caregiver Group * Child Age 8-12	-2.076	1.486	-1.397	0.163	-4.992	0.840	0.002
	DMD Caregiver Group * Child Age 2-7	0[a]						
	Comparison Caregiver Group * Child Age 18+	0[a]						
	Comparison Caregiver Group * Child Age 13-17	0[a]						
	Comparison Caregiver Group * Child Age 8-12	0[a]						
	Comparison Caregiver Group * Child Age 2-7	0[a]						
**PROMIS Mental**	Intercept	49.956	1.111	44.952	0.000	47.775	52.137	0.645
	Propensity Score	-1.109	1.745	-0.636	0.525	-4.532	2.314	0.000
	Child Age 18+	0.490	1.081	0.453	0.650	-1.631	2.611	0.000
	Child Age 13-17	2.759	1.101	2.505	0.012	0.598	4.919	0.006
	Child Age 8-12	0.637	1.006	0.633	0.527	-1.337	2.611	0.000
	Child Age 2-7	0[a]						

DMD Caregiver Group	3.138	1.080	2.904	0.004	1.018	5.258	0.008
Comparison Caregiver Group	0[a]						
DMD Caregiver Group * Child Age 18+	-4.491	1.497	-3.001	0.003	-7.428	-1.554	0.008
DMD Caregiver Group * Child Age 13-17	-8.991	1.561	-5.759	0.000	-12.055	-5.928	0.029
DMD Caregiver Group * Child Age 8-12	-4.218	1.499	-2.814	0.005	-7.159	-1.277	0.007
DMD Caregiver Group * Child Age 2-7	0[a]						
Comparison Caregiver Group * Child Age 18+	0[a]						
Comparison Caregiver Group * Child Age 13-17	0[a]						
Comparison Caregiver Group * Child Age 8-12	0[a]						
Comparison Caregiver Group * Child Age 2-7	0[a]						

**NeuroQOL Pos.Affect**	Intercept	54.937	0.805	68.283	0.000	53.358	56.515	0.807
	Propensity Score	-0.685	1.263	-0.543	0.587	-3.163	1.793	0.000
	Child Age 18+	0.500	0.782	0.639	0.523	-1.035	2.036	0.000
	Child Age 13-17	0.652	0.797	0.818	0.414	-0.912	2.216	0.001
	Child Age 8-12	0.760	0.728	1.044	0.297	-0.669	2.189	0.001
	Child Age 2-7	0[a]						
	DMD Caregiver Group	-0.492	0.782	-0.628	0.530	-2.026	1.043	0.000
	Comparison Caregiver Group	0[a]						
	DMD Caregiver Group * Child Age 18+	-2.409	1.084	-2.223	0.026	-4.535	-0.283	0.004
	DMD Caregiver Group * Child Age 13-17	-1.460	1.130	-1.292	0.197	-3.678	0.758	0.001
	DMD Caregiver Group * Child Age 8-12	-0.148	1.085	-0.136	0.892	-2.277	1.981	0.000
	DMD Caregiver Group * Child Age 2-7	0[a]						
	Comparison Caregiver Group * Child Age 18+	0[a]						
	Comparison Caregiver Group * Child Age 13-17	0[a]						
	Comparison Caregiver Group * Child Age 8-12	0[a]						
	Comparison Caregiver Group * Child Age 2-7	0[a]						
**Ryff Envr. Mastery**	Intercept	28.648	0.814	35.193	0.000	27.051	30.246	0.527
	Propensity Score	2.209	1.278	1.728	0.084	-0.299	4.716	0.003
	Child Age 18+	2.791	0.792	3.525	0.000	1.238	4.344	0.011
	Child Age 13-17	1.887	0.807	2.340	0.019	0.304	3.469	0.005
	Child Age 8-12	0.849	0.737	1.152	0.250	-0.597	2.295	0.001
	Child Age 2-7	0[a]						
	DMD Caregiver Group	2.678	0.791	3.384	0.001	1.125	4.231	0.010

	Comparison Caregiver Group	0[a]						
	DMD Caregiver Group * Child Age 18+	-5.464	1.096	-4.983	0.000	-7.615	-3.312	0.022
	DMD Caregiver Group * Child Age 13-17	-6.112	1.144	-5.344	0.000	-8.356	-3.868	0.025
	DMD Caregiver Group * Child Age 8-12	-3.361	1.098	-3.061	0.002	-5.515	-1.207	0.008
	DMD Caregiver Group * Child Age 2-7	0[a]						
	Comparison Caregiver Group * Child Age 18+	0[a]						
	Comparison Caregiver Group * Child Age 13-17	0[a]						
	Comparison Caregiver Group * Child Age 8-12	0[a]						
	Comparison Caregiver Group * Child Age 2-7	0[a]						
**Resilience**	Intercept	-0.286	0.122	-2.342	0.019	-0.525	-0.046	0.005
	Propensity Score	0.674	0.192	3.517	0.000	0.298	1.050	0.011
	Child Age 18+	0.008	0.119	0.069	0.945	-0.225	0.241	0.000
	Child Age 13-17	0.068	0.121	0.566	0.572	-0.169	0.306	0.000
	Child Age 8-12	0.028	0.110	0.254	0.800	-0.189	0.245	0.000
	Child Age 2-7	0[a]						
	DMD Caregiver Group	-0.068	0.119	-0.571	0.568	-0.300	0.165	0.000
	Comparison Caregiver Group	0[a]						
	DMD Caregiver Group * Child Age 18+	0.108	0.164	0.659	0.510	-0.214	0.431	0.000
	DMD Caregiver Group * Child Age 13-17	-0.299	0.171	-1.747	0.081	-0.636	0.037	0.003

DMD Caregiver Group * Child Age 8-12	0.000	0.165	0.003	0.998	-0.322	0.323	0.000
DMD Caregiver Group * Child Age 2-7	0[a]						
Comparison Caregiver Group * Child Age 18+	0[a]						
Comparison Caregiver Group * Child Age 13-17	0[a]						
Comparison Caregiver Group * Child Age 8-12	0[a]						
Comparison Caregiver Group * Child Age 2-7	0[a]						

**Stress**	Intercept	43.564	1.660	26.244	0.000	40.307	46.821	0.382
	Propensity Score	-10.851	2.606	-4.164	0.000	-15.963	-5.738	0.015
	Child Age 18+	-7.513	1.614	-4.654	0.000	-10.680	-4.345	0.019
	Child Age 13-17	-5.636	1.645	-3.427	0.001	-8.863	-2.409	0.010
	Child Age 8-12	-1.162	1.503	-0.773	0.439	-4.110	1.786	0.001
	Child Age 2-7	0[a]						
	DMD Caregiver Group	-5.700	1.614	-3.532	0.000	-8.867	-2.534	0.011
	Comparison Caregiver Group	0[a]						
	DMD Caregiver Group * Child Age 18+	10.575	2.236	4.730	0.000	6.188	14.962	0.020
	DMD Caregiver Group * Child Age 13-17	15.597	2.332	6.688	0.000	11.021	20.172	0.039
	DMD Caregiver Group * Child Age 8-12	3.897	2.239	1.741	0.082	-0.496	8.289	0.003
	DMD Caregiver Group * Child Age 2-7	0[a]						
	Comparison Caregiver Group * Child Age 18+	0[a]						
	Comparison Caregiver Group * Child Age 13-17	0[a]						
	Comparison Caregiver Group * Child Age 8-12	0[a]						
	Comparison Caregiver Group * Child Age 2-7	0[a]						
**Difficulty paying bills**	Intercept	2.363	0.138	17.186	0.000	2.093	2.633	0.210
	Propensity Score	-0.809	0.216	-3.746	0.000	-1.232	-0.385	0.012
	Child Age 18+	-0.402	0.134	-3.003	0.003	-0.664	-0.139	0.008
	Child Age 13-17	-0.296	0.136	-2.171	0.030	-0.563	-0.028	0.004
	Child Age 8-12	-0.060	0.124	-0.486	0.627	-0.305	0.184	0.000
	Child Age 2-7	0[a]						
	DMD Caregiver Group	0.015	0.134	0.113	0.910	-0.247	0.277	0.000

Comparison Caregiver Group	0[a]						
DMD Caregiver Group * Child Age 18+	0.525	0.185	2.835	0.005	0.162	0.888	0.007
DMD Caregiver Group * Child Age 13-17	0.881	0.193	4.563	0.000	0.502	1.260	0.018
DMD Caregiver Group * Child Age 8-12	0.258	0.185	1.389	0.165	-0.106	0.621	0.002
DMD Caregiver Group * Child Age 2-7	0[a]						
Comparison Caregiver Group * Child Age 18+	0[a]						
Comparison Caregiver Group * Child Age 13-17	0[a]						
Comparison Caregiver Group * Child Age 8-12	0[a]						
Comparison Caregiver Group * Child Age 2-7	0[a]						

a. This parameter is set to zero because it is redundant.

Between-Subjects Factors

Parameter Estimates

**Dependent Variable: Hours Miss**	**Value Label**	**N**			**95% Confidence Interval**		**Partial**
**Parameter**	**B**	**Std. Error**	**t**	**Sig.**	**Lower Bound**	**Upper Bound**	**Eta**
Intercept	-5.40	1.15	5.50	0.000	4.06	8.57	0.039
Propensity Score	-2.16	1.89	-2.85	0.004	-9.11	-1.68	0.011
Child Age 18+	-1.03	1.20	-1.80	0.072	-4.51	0.19	0.004
Child Age 13-17	-0.42	1.09	-0.95	0.341	-3.17	1.10	0.001
Child Age 8-12	0[a]	1.00	-0.42	0.678	-2.38	1.55	0.000
Child Age 2-7	3.28						
DMD Caregiver Group	0[a]	1.13	2.90	0.004	1.06	5.50	0.011
Comparison Caregiver Group	-0.77						

DMD Caregiver Group * Child Age 18+	-2.37	1.65	-0.47	0.642	-4.01	2.47	0.000
DMD Caregiver Group * Child Age 13-17	-2.25	1.76	-1.35	0.178	-5.83	1.08	0.002
DMD Caregiver Group * Child Age 8-12	0[a]	1.62	-1.39	0.166	-5.43	0.93	0.003
DMD Caregiver Group * Child Age 2-7	0[a]						
Comparison Caregiver Group * Child Age 18+	0[a]						
Comparison	0[a]						
Comparison Caregiver Group * Child Age 8-12	0[a]						
Comparison Caregiver Group * Child Age 2-7							

a. This parameter is set to zero because it is redundant.
