# Supplementary material for: Characterizing the quality-of-life impact of Duchenne muscular dystrophy on caregivers: a case-control investigation
Source: J Patient Rep Outcomes. 2021 Nov 20;5:124. doi: 10.1186/s41687-021-00386-y (PMC8605451; doi:10.1186/s41687-021-00386-y)
Supplement: Supplementary file 5 — Additional file 5. Supplemental Figure 1. Bar chart showing numbers of DMD and comparison caregivers in the four Child Age Group strata. [file 41687_2021_386_MOESM5_ESM.pdf]

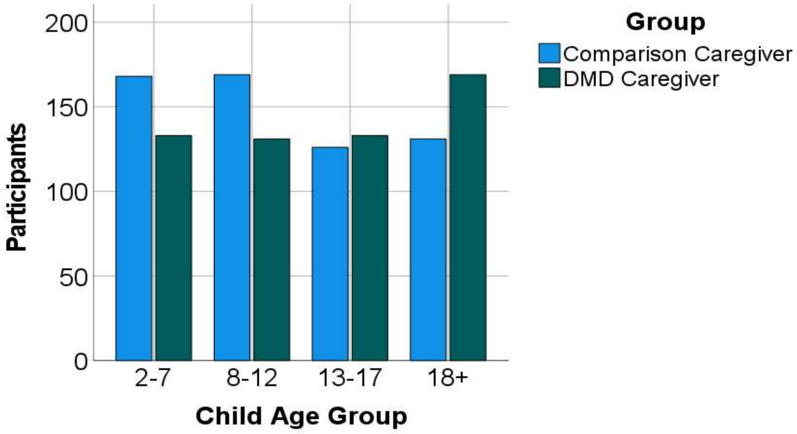

Supplemental Figure 1. Bar chart showing numbers of DMD and comparison caregivers in the four Child Age Group strata. The study sample included 566 DMD caregivers and 594 comparison caregivers, representing nearly equally the four Child Age Group strata.
